# Energy‐dependent OAR sparing and dose conformity for total marrow irradiation of obese patients

**DOI:** 10.1002/acm2.12413

**Published:** 2018-08-11

**Authors:** Amanda J. Cherpak, Thalat Monajemi, Krista Chytyk‐Praznik, Liam Mulroy

**Affiliations:** ^1^ Nova Scotia Cancer Centre Nova Scotia Health Authority Halifax NS Canada; ^2^ Department of Radiation Oncology Dalhousie University Halifax NS Canada; ^3^ Department of Physics and Atmospheric Science Dalhousie University Halifax NS Canada

**Keywords:** OAR sparing, total marrow irradiation, treatment planning, volumetric arc therapy (VMAT)

## Abstract

**Purpose:**

To investigate the effect on target coverage and organs at risk sparing by using 10 versus 6 MV for VMAT total marrow irradiation of obese patients.

**Methods and Materials:**

Twenty‐six total marrow irradiation, TMI, treatment plans delivered between December 2014 and June 2017 were reviewed and 10 were chosen for replanning based on patient characteristics and plan metrics. Beam geometry and isocenter placement were conserved, energy was changed from 6 to 10 MV and plans were reoptimized. Resulting dose distributions were compared to original plans to evaluate any potential advantage of choosing one energy over the other.

**Results:**

Target coverage and total monitor units were consistent between the 6 and 10 MV plans when averaged over all ten patients. Improvement in the conformity index (−11.0%, *P* = 0.009) when using 10 MV was statistically significant compared to the 6 MV plans. Volumes of normal tissue receiving 50%, 75%, and 90% Rx all decreased for the 10 MV plans compared to the original 6 MV plans. The mean dose to individual OARs decreased significantly for all investigated structures except for the lenses, oral cavity, and genitalia. The largest decreases in D_mean_ were found for the rectum (22.4%, *P *= 0.004) and bladder (18.1%, *P* = 0.005). The three highest priorities for sparing during plan optimization (lungs, liver, and heart), showed decreases of 7.6%, 16.1%, and 13.0%.

**Conclusions:**

Use of a higher energy 10 MV beam provided similar dose to target while achieving increased OAR and normal tissue sparing for the patients reviewed in this study.

## 1 INTRODUCTION

Total body irradiation, TBI, has long been used as part of a conditioning regime prior to bone marrow or peripheral stem cell transplant for patients with hematologic malignancies such as leukemia, lymphoma, and multiple myeloma.^1^, ^2^ The purpose of the treatment is two‐fold; first, to ablate the malignant cancer cells and second, to suppress the immune system and increase the chance of acceptance of donor marrow.^1^ Traditional techniques of delivering TBI use large open fields and accessories such as body compensators, lung attenuators, and beam spoilers to achieve a dose homogeneity of ±10% over the entire body.^1^, ^3^, ^4^, ^5^ Randomized trials have demonstrated the effectiveness of TBI conditioning regimes prior to bone marrow transplant.^6^, ^7^, ^8^, ^9^ However, due to the high dose to both the bone marrow and normal healthy tissues, toxicities and treatment‐related mortalities are a significant concern.^10^, ^11^ Side effects from TBI can be extensive and include acute symptoms such as nausea and loss of appetite as well as infertility and secondary malignancies.^12^, ^13^

Advanced treatment planning and linac capabilities coupled with intrafractional imaging have recently made it possible to target only the bone marrow using a technique called total marrow irradiation (TMI).^14^, ^15^, ^16^, ^17^ TMI treatments are able to reduce the dose to organs at risk, OARs, to 15–65% of the prescription dose rather than 90–110% as given in TBI treatments.^18^ Depending on desired planning target volume, PTV, coverage (85%Rx vs 95%Rx), OAR sparing could potentially be further reduced by another 4–51%.^17^ Evidence has shown that focusing the dose on the patient's bone marrow rather than the entire body and decreasing dose to healthy tissue can lead to minimization of toxicities such as mucositis, vomiting, and diarrhea.^18^, ^19^, ^20^

Our center has recently implemented a TMI protocol that includes a combination of VMAT fields to treat the bone marrow from head to approximately mid‐thigh and extended source‐to‐surface distance, SSD, parallel‐opposed pair, POP, fields to treat the rest of the legs.^21^ Adequate PTV coverage was achieved for all patients, however, some treatment plans were particularly challenging to optimize due to large patient anatomy. In these cases, it was often necessary to accept larger than average hot spots V(110%) > 40%, higher mean dose to OARs, or increased monitor units. The PTV spans the length of the entire body so in an average person, target depth can range from a few millimeters at the anterior portion of the ribs to more than 10 cm in the pelvis. In some of the larger patients treated, target depth was as much as 18 cm. All treatment plans to date have used a beam energy of 6 MV, however, the retrospective planning study presented in this manuscript aims to investigate the effects of using a 10 MV beam for TMI VMAT fields in comparison to 6 MV. High energy beams (10–18 MV) have historically been a part of TBI treatments, particularly for patients of thickness greater than 35 cm;^3^, ^22^ however, published experience with modulated TMI fields has been limited to 6 MV IMRT and VMAT or helical tomotherapy.^14^, ^15^, ^23^, ^24^, ^25^, ^26^, ^27^, ^28^ Potential disadvantages of increasing beam energy include larger penumbra and the production of secondary neutrons originating in the head of the linac. These neutrons can contribute to integral dose for energies greater than 10 MV, therefore this study will not escalate beam energy beyond this level.^29^, ^30^, ^31^, ^32^ This work explores the hypothesis that a more penetrating beam may be useful in achieving target coverage while increasing healthy tissue sparing for certain TMI patients. Initial efforts will focus specifically on clinically obese patients, which generally represent cases of increased separation and depth of target.

## 2 MATERIALS AND METHODS

Patients were scanned in the head‐first supine position in a S‐frame thermoplastic mask and an indexable vaclok that extended to approximately midthigh. Arms were tight at the patient's side and secured with a Velcro body strap. Bolus sleeves of 0.5 cm thickness were used around the arms from below the elbow to the wrist. Due to physical limitations of the CT and treatment couches, a second CT scan was done with patients in the feet‐first supine position. A large bolus sheet of 1 cm thickness was used over the lower legs from above the knees to the ankles. The TMI clinical target volume, CTV, consisted of the brain, spinal cord, and skeletal bones extending from the vertex of the skull to middle of the thighs excluding the mandible, nasal bones, and small bones of the hands below the wrist. As described in Ayodogan et al., contouring entire bones allows for a generous margin around the bone marrow.^17^ The PTV was not created from a uniform expansion of the CTV, but rather was drawn to encompass the CTV plus adjacent soft tissues (e.g., intercostal tissues, soft tissues between radius and ulna, soft tissues between scapulae and posterior chest wall) similar to other groups.^26^ Therapists reported difficulty in positioning the arms during patient treatments so a radial expansion of 5 mm was used for the arm bones from the surgical neck of humerus to the inferior aspect of radius and ulna to allow for a greater setup margin.

The prescription was 12 Gy, delivered in six fractions of 2 Gy each. Patients were treated twice a day, with a minimum of 6 h between treatments. All patients were treated using a 6 MV beam on a Clinic 2100iX linac. Treatment consisted of a combination of VMAT and extended SSD POP fields planned using PRO version 11 and Eclipse AAA version 11 (Varian, Palo Alto, CA). The VMAT fields treated the bone marrow from head to pelvis with the patient positioned head‐first supine on the treatment couch. The inferior edge of the VMAT‐treated target depended on patient height and physical limits of couch extension. A total of ten arcs were used, spread over five to six isocenters along the cranial‐caudal direction. Patient setup was verified before delivery of each set of arcs using orthogonal kilovoltage images (anterior–posterior and right–left) and matching to bone. When the arms obscured the view of the spine (from the shoulders to abdomen), orthogonal images were taken at oblique angles of 315° and 45°. Workflow consisted of shifting to an isocenter, imaging, applying any necessary shifts or adjustments to patient position, then delivery of the arcs. This process was repeated for all isocenters. The patient was then repositioned feet‐first supine and POP fields were used to treat the rest of the legs. Two sets of POPs, junctioned just below the knee were typically necessary to cover the full extent of the patients’ legs. In cases where the superior edge of the POP fields was near genitalia, MLC shielding was added to limit dose to OARs. Anterior–posterior megavoltage images were taken for position verification before treatment of each set of beams.

During treatment planning, the POP beams were created first and the resulting dose distribution was used as a base plan during VMAT optimization. This allowed for the optimizer to smooth out the junction region between the two sets of plans and avoid hot spots. The entire PTV was to be covered by 90% of the prescription (V(10.8 Gy) > 99%). Although not a part of the initial planning criteria, experience showed that many plans could achieve V(12 Gy) of 95% or greater so this also became a useful benchmark. Other planning goals with a high priority included 90% dose coverage of the entire PTV and mean dose to the lungs, heart and liver of less than 70% (D_mean_ < 8.4 Gy). Other OARs contoured and used in plan optimization included eyes, parotids, esophagus, midline mucosa, kidneys, bowel space, bladder, rectum, and genitalia.

The twenty‐six treatment plans delivered between December 2014 and June 2017 were reviewed and patient characteristics and plan quality metrics of the original 6 MV plans were compared to determine which ones should be included in this study. To identify which patients may benefit from a more penetrating beam, the following measurements were noted: width (left–right) at shoulders, width (left–right and anterior–posterior) at elbows, total volume of body contour, and body mass index, BMI. Ten of the original twenty‐six patients were classified as obese (BMI > 30 kg/m^2^). The BMI among this group of ten was on average 36.7 ± 6.6 kg/m^2^ and ranged as high as 48.5 kg/m^2^. Eight of these ten patients had all width and volume measurements above the average for all twenty‐six patients. The VMAT plans for these eight patients all had total plan MU's that were higher than the group average ((MU_Patient(x)_ − MU_Average_)/MU_Average_ ranged from 2% to 46%). All plans had the PTV covered by 90% of the prescription dose, 10.8 Gy, however, the plans for most of the obese patients resulted in higher OAR doses and larger hot spot volumes. In the end, all ten patients that fit the clinical definition of obesity were chosen for replanning to explore if a higher energy beam would improve the plans beyond what was initially achieved. The remaining sixteen TMI patients that have been treated at our center were not included in this study.

To create the new 10 MV VMAT plans, beam geometry was copied from the original 6 MV plan for each patient. Collimator angles, field sizes, as well as number and placement of isocenters were conserved. Energy was changed from 6 to 10 MV and plans were reoptimized. Due to availability of commissioned beam energies, 10 MV plans were calculated for a TrueBeam linac with a similar field size and MLC configuration as the original linac. Optimization objectives were similar to those used in the original plans, with optimization structures and constraints revised as planning progressed to achieve coverage and reduce hot spots. The original 6 MV plans were created by a group of five physicists, and a subset of three of these physicists completed the 10 MV replanning. To keep methods consistent, plans were developed with the following priorities: (a) achieve PTV coverage: V(10.8 Gy) > 99%, V(12 Gy) > 90%, (b) reduce magnitude of V(13.2 Gy) to <40%, if possible, (c) reduce mean dose to lungs, liver, and heart if greater than 70%Rx. As it was noted that V(12 Gy) > 95% could often be achieved for the 6 MV plans, the physicist would continue optimizing within reason to try and reach this goal for the 10 MV plans. Care was taken to not significantly exceed total MUs used in the 6 MV plan so as not to give the 10 MV plans an unfair advantage for dosimetric indices due to increased modulation. Physicists were all experienced in TMI planning and stopped when a clinically acceptable plan was achieved within reasonable time and effort.

The following parameters were calculated to compare dose to normal tissue between the 6 and 10 MV plans for each patient: D_mean_ for OARs and volume of normal tissue receiving 50% (6 Gy), 75% (9 Gy), and 90% (10.8 Gy) of the prescription dose. A structure was created out of the specified isodose curve and a Boolean operator was used to subtract the PTV volume from that structure (leaving only normal tissue receiving the specific dose) and called “Body‐PTV”. These volumes are referred to as V(6 Gy)_Body‐PTV_, V(9 Gy)_Body‐PTV_, and V(10.8 Gy)_Body‐PTV_. PTV coverage was assessed using V(10.8 Gy)_PTV_, V(12 Gy)_PTV_, conformity index (CI), and homogeneity index (HI). The conformity index, CI, is a measure of how much the prescription isodose conforms to the PTV rather than spill into normal tissue. It was calculated according to ICRU report 62 as the ratio between the volume of the body receiving a selected dose (12 Gy) and the volume of PTV receiving that same dose, V(12 Gy)_Body_/V(12 Gy)_PTV._ 33 The homogeneity index, HI, is a measure of the standard deviation in dose to the PTV and is calculated as a ratio of the maximum and minimum dose to the entire target volume.^33^ In this case, maximum dose is defined as the dose to the hottest 5% of the volume, D(5%), and the minimum dose is defined as the dose to the coldest 95% of the volume, D(95%), from the PTV DVH. For both CI and HI, the ideal value is 1 and the values will increase as conformity and homogeneity decrease.

## 3 RESULTS

Planning results are shown in Tables 1 and 2. For each parameter compared, the mean and standard deviation of the mean $(\overset{¯}{x}\pm \sigma_{\overset{¯}{x}}),$ and the percent increase from 6 to 10 MV are given. These values were averaged over all patient plans for the given energy. Percent difference was calculated as $\left[{\left(\bar{D_{\mathrm{mean}}}\right)}_{10MV}-{\left(\bar{D_{\mathrm{mean}}}\right)}_{6MV}\right]/\left[{\left(\bar{D_{\mathrm{mean}}}\right)}_{6MV}\right]$ therefore a negative value for “percent difference” means the value decreased for the 10 MV plan compared to the 6 MV plan. A paired Student t‐test and power analysis were performed for each parameter to determine the statistical significance of any differences found.^29^ A *P*‐value less than or equal to 0.05 was considered statistically significant. Table 1 shows comparisons of dose coverage and PTV statistics. Although some subtle differences could be seen between the individual patient plans for the two energies, there were no statistically significant differences between the 6 and 10 MV plans for HI, total monitor units, V(12 Gy)_PTV_, V(13.2 Gy)_PTV_, or maximum dose, D(2%)_PTV_, when averaged over the total patient population. Improvement in the conformity index (−11.0%, *P* = 0.009) when using 10 MV was significant compared to the 6 MV plans. The difference in V(10.8 Gy)_PTV_ (0.1%, *P* = 0.046) was statistically significant but clinically irrelevant (see Table 1). It was also found that the volumes of normal tissue receiving 50%, 75%, and 90% Rx all decreased for the 10 MV plans compared to the original 6 MV plans.

**Table 1:** Results of 10 MV plans compared to original 6 MV plans for the ten obese patients chosen for replanning. Values are averaged over all ten patients. For each category, mean and standard deviation of the mean, percent difference, and *P*‐value are shown. Percent difference is calculated as $\left[{\left(\bar{D_{\mathrm{mean}}}\right)}_{10MV}-{\left(\bar{D_{\mathrm{mean}}}\right)}_{6MV}\right]/\left[{\left(\bar{D_{\mathrm{mean}}}\right)}_{6MV}\right]$

Category	$\overset{¯}{x}\pm \sigma_{\overset{¯}{x}}$		Percent difference	*P*‐value
	6 MV	10 MV		
Conformity index	1.70 ± 0.29	1.51 ± 0.19	−11.0%	**0.009**
Homogeneity index	1.19 ± 0.06	1.17 ± 0.02	−1.7%	0.159
Total monitor units	6000 ± 1100	5622 ± 934	−5.9%	0.081
V(10.8 Gy)_PTV_	99.64 ± 0.28%	99.76 ± 0.25%	0.1%	**0.046**
V(12 Gy)_PTV_	93.7 ± 1.9%	94.7 ± 1.2%	1.0%	0.206
V(13.2 Gy)_PTV_	37 ± 19%	35 ± 15%	−14.8%	0.137
D(2%)_PTV_	14.39 ± 0.62 Gy	14.39 ± 0.41 Gy	0.0%	0.997
[V(6 Gy)_Body‐PTV_]/10^3^	54.5 ± 9.8 cm^3^	53.1 ± 9.9 cm^3^	−7.6%	**0.001**
[V(9 Gy)_Body‐PTV_]/10^3^	32.1 ± 7.0 cm^3^	27.4 ± 6.6 cm^3^	−14.5%	**0.001**
[V(10.8 Gy)_Body‐PTV_]/10^3^	16.6 ± 4.7 cm^3^	13.1 ± 4.2 cm^3^	−21.1%	**0.002**

**Table 2:** Average mean dose to organs at risk for 10 MV plans compared to original 6 MV plans. Values are averaged over all ten patients chosen for replanning. For each OAR, percent difference and *P*‐value are also shown. Percent difference is calculated as $\left[{\bar{D}}_{\mathrm{mean}}\left(10\;\mathrm{MV}\right)-{\bar{D}}_{\mathrm{mean}}\left(6\;\mathrm{MV}\right)\right]/{\bar{D}}_{\mathrm{mean}}\left(6\;\mathrm{MV}\right)$

Organ at risk	${\bar{D}}_{\mathrm{mean}}\pm \sigma_{{\bar{D}}_{\mathrm{mean}}}\left(Gy\right)$		Percent difference	*P*‐value
	6 MV	10 MV		
Lungs	7.9 ± 0.4 Gy	7.3 ± 0.3 Gy	−7.6%	**0.001**
Liver	7.8 ± 0.6 Gy	6.5 ± 1 0.4 Gy	−16.1%	**0.000**
Heart	6.6 ± 0.8 Gy	5.7 ± 0.3 Gy	−13.0%	**0.002**
Kidney(L)	7.3 ± 0.7 Gy	6.5 ± 1 0.5 Gy	−11.0%	**0.015**
Kidney(R)	7.4 ± 1.0 Gy	6.5 ± 0.7 Gy	−12.2%	**0.003**
Lens(L)	5.0 ± 0.7 Gy	4.7 ± 1 0.4 Gy	−5.2%	0.283
Lens(R)	5.0 ± 1.0 Gy	4.7 ± 0.4 Gy	−6.4%	0.172
Midline mucosa	6.7 ± 0.9 Gy	5.7 ± 1 0.3 Gy	−15.4%	**0.006**
Oral cavity	4.3 ± 1.3 Gy	4.0 ± 0.6 Gy	−6.5%	0.102
Parotid(L)	5.7 ± 1.0 Gy	5.2 ± 0.4 Gy	−9.7%	**0.005**
Parotid(R)	5.9 ± 0.7 Gy	5.0 ± 0.3 Gy	−14.6%	**0.026**
Rectum	7.0 ± 1.3 Gy	5.4 ± 0.8 Gy	−22.4%	**0.004**
Bladder	6.5 ± 1.0 Gy	5.4 ± 0.6 Gy	−18.1%	**0.005**
Bowel space	7.6 ± 0.7 Gy	6.4 ± 0.6 Gy	−15.5%	**0.000**
Esophagus	8.1 ± 0.5 Gy	7.8 ± 0.4 Gy	−4.3%	**0.040**
Genitalia	3.2 ± 1.3 Gy	3.0 ± 1.3 Gy	−6.9%	0.524

Table 2 shows that the mean dose to all OARs was lower for the 10 MV plans. These differences were found to be statistically significant (*P* < 0.05) in all cases except for the lenses, oral cavity and genitalia. The largest decreases in D_mean_ were found for the rectum (22.4%, *P* = 0.004) and bladder (18.1%, *P* = 0.005). The three highest priorities for sparing during plan optimization (lungs, liver, and heart), showed decreases of 7.6%, 16.1%, and 13.0% respectively and this behavior was consistent among the individual patients.

## 4 DISCUSSION

The parameters shown in the first seven rows of Table 1 define magnitude and homogeneity of dose to the PTV and degree of modulation in the plan. Since these values are not any worse than for the 6 MV plans, it can be assumed that any additional sparing of healthy tissues found between the two sets of plans does not come at the cost of compromised dose to target or overall plan quality. One challenge with TMI planning for obese patients is that total monitor units can rise very quickly when trying to achieve target coverage and organ sparing similar to smaller patients by increasing modulation. Total plan monitor units were lower for individual 10 MV plans in all but one case, where the difference was 7%. For the rest of the patient plans, monitor units were decreased by 2–24% compared to the 6 MV plans. The decrease in monitor units for the 10 MV plans indicates that the higher energy beams were able to achieve equivalent target dose coverage more efficiently than the lower energy beams. This reduction in MUs for higher energy beams agrees with results found by other groups that have compared 6 MV with 10, 15, and 18 MV VMAT and IMRT plans for prostate and cervix cancer cases.^34^, ^35^, ^36^

**Figure 1:**
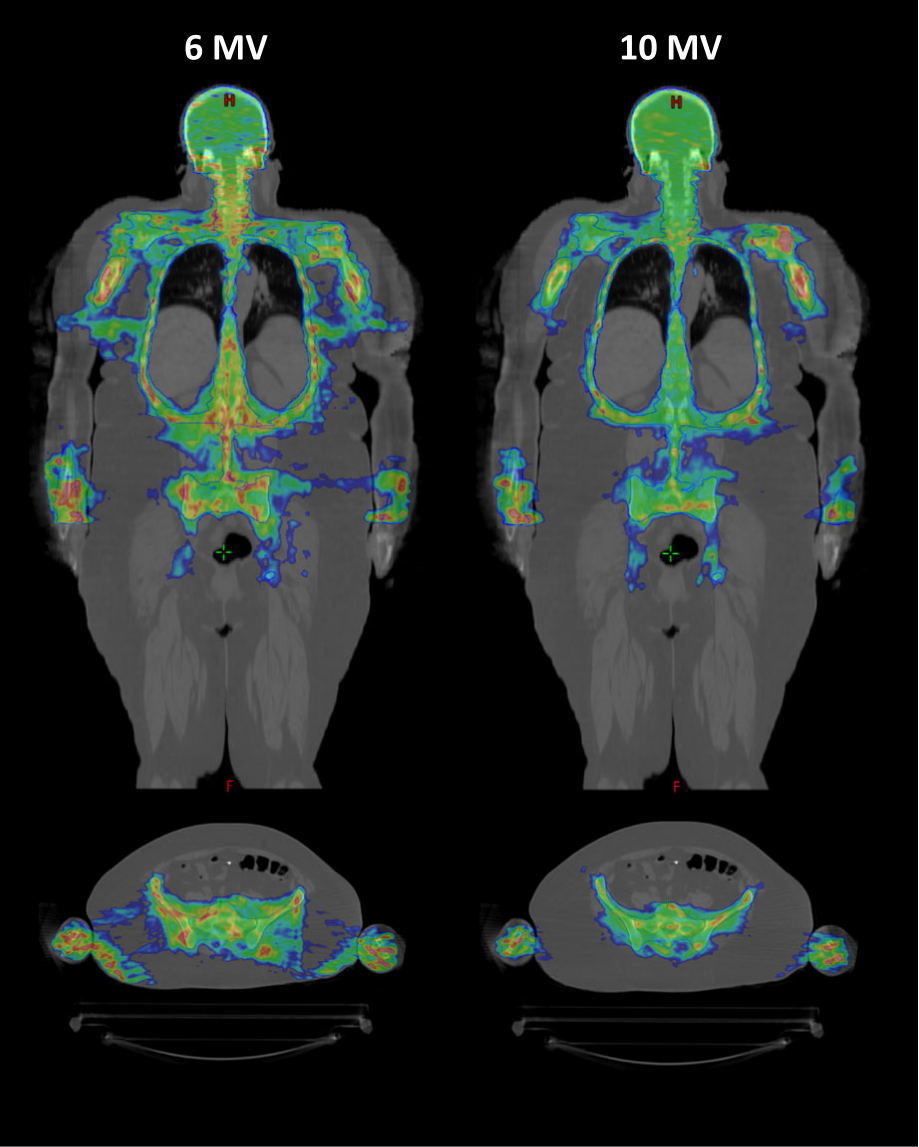
Comparison of 90–120% isodose distributions for 6 and 10 MV for Patient 8.

The 10 MV plans resulted in similar V(13.2 Gy) values compared to the original plans, so a decrease in hot spots was not noted. A decrease was seen, however, in the normal tissue dose volumes (V(6 Gy)_Body‐PTV,_ V(9 Gy)_Body‐PTV,_ V(10.8 Gy)_Body‐PTV_). This behavior is also noted with the improvement seen for conformity index, which reflects the nature of the volume (PTV vs healthy tissue) receiving 100% of the prescription dose. An example of a specific patient plan can be seen in Fig. 1. The dose splash outside of the PTV is a common feature of TMI plans for obese patients. This work demonstrates that this can be significantly improved when using a higher energy beam. For the patients reviewed in this study, the higher energy treatment was able to better exploit the conformal nature of TMI treatment.

Dose to OARs was consistently more favorable for the 10 MV plans. Plans using 10 MV resulted in a reduced mean dose to lungs of −7.3%, as seen in Table 2. While this decrease is not as large as for some other OARs, it does have implications on the plan as a whole since adequate lung sparing was often difficult to achieve. During the original planning of the cases used in this study, compromises were sometimes made in terms of target dose homogeneity or increased monitor units in order to get the mean lung dose below 70% Rx (8.4 Gy) since this is the organ‐at‐risk with the highest priority. Aside from decreasing potential side effects like pneumonitis, improved lung sparing can therefore potentially allow for improving other plan metrics or decreasing overall planning time as goals are more quickly met.

Dose to the genitalia is affected by the patient height, as some plans required this region to be covered by the POP beams with the patient in the feet‐first position while in other plans this region was fully encompassed by the VMAT arcs. Mean dose is therefore not limited to the effect of only the 10 MV VMAT fields. No difference in mean dose was seen either for the lenses or oral cavity. Depth of the targets in the head and neck were generally unaffected by differences in BMI or overall size, so these values did not differ from the rest of the patient group to begin with when using the 6 MV beams. An energy comparison for prostate bed irradiation found the largest reduction in dose when using a higher energy beam to be for the more shallow OARs (femoral heads) compared to more deep‐seated ones.^29^ This could potentially be due to the fact that they were comparing dose to OARs that were all in proximity to a localized target (i.e., prostate bed). The depth of the TMI target varies throughout the body so comparison of shallow OARs in the head and neck region to those found deeper in the abdomen and pelvis may not be as meaningful since their position relative to the target is so different. One concern before planning with the 10 MV beam was whether dose coverage of the skull would be more difficult than for 6 MV, since the PTV becomes very superficial in this area. This did not end up being a problem and it was encouraging to note that there was no increase in dose to organs of this area. A potential future option to explore may be to use a mixed energy approach, with a 6 MV beam used to cover the head and neck and 10 MV used for the rest of the body where excess adipose tissue is normally found.

It should be noted that while the 10 MV plans were created by a subset of physicists who had prepared the 6 MV plans, only the latter were created within the confines of clinical time constraints. The 10 MV plans were created from the basis of the 6 MV plans (isocenter location, number of beams, etc.) and similar optimization goals were applied. It could be argued that since the 10 MV plans did not have higher MUs (which increase with additional runs of the optimization), they did not benefit from additional optimization/planning.

## 5 CONCLUSION

In this study, we compare 6 and 10 MV TMI plans for a selected group of patients that were chosen based on BMI. Dose coverage and homogeneity for the PTV were equivalent for the 6 and 10 MV plans, as was the volume receiving 110%Rx (13.2 Gy). A statistically significant decrease in the mean dose to all organs at risk was achieved for the 10 MV plans except for the genitalia, lenses, and oral cavity. Decreases in dose to lungs, liver, and heart were considered to be most important as these OARs often drive the optimization once target coverage is achieved. The conformity of high dose was improved with the use of the higher energy beams as seen in results for the conformity index and normal tissues dose regions. These gains also came with a decrease in total monitor units. Patients’ bodies vary greatly in dimension and shape and there may be further subtleties to choosing which energy would be best that were not explored here. Future work could potentially include broadening the study to include all TMI patients to further investigate the limits of the benefits reported here. The motivation for studying this particular group of patients was the difficulty encountered during preparation of the original plans compared to plans of smaller patients. Clinical experience could potentially lead to anticipation of such challenges when reviewing patient anatomy and perhaps drive the use of a higher energy beam from the beginning. What this work does show, however, is that the use of 10 MV can provide superior sparing of healthy tissues for patients that are clearly larger than average and can provide a useful alternative when encountering challenges with a 6 MV plan if energy choice is not immediately obvious.

## CONFLICT OF INTEREST

The authors declare no conflict of interest.
